# MicroRNAs in Colorectal Cancer Immunotherapy: Biomarkers, Resistance Mechanisms, and Strategies to Convert “Cold” Tumors to “Hot”

**DOI:** 10.3390/genes17091025

**Published:** 2026-08-28

**Authors:** Jin Yan, Ruixia Ma, Yaguang Xi

**Affiliations:** 1 Department of Pharmaceutical and Biomedical Sciences, College of Pharmacy, University of Georgia, Athens, GA 30602, USA; 2 Innovations in Drug Discovery (IDD) Program, College of Pharmacy, University of Georgia, Athens, GA 30602, USA

**Keywords:** microRNA, colorectal cancer, immune checkpoint inhibitors, tumor microenvironment, extracellular vesicles, biomarkers

## Abstract

Colorectal cancer (CRC) remains a major cause of cancer morbidity and mortality. Immune checkpoint inhibitors (ICIs) have transformed the treatment of microsatellite instability-high/mismatch repair-deficient (MSI-H/dMMR) CRC, yet most CRCs are microsatellite-stable/mismatch repair-proficient (MSS/pMMR) and remain poorly responsive to immunotherapy. MicroRNAs (miRNAs) are well positioned to influence this biology because individual miRNAs can coordinate multiple tumor-intrinsic and microenvironmental programs that shape antitumor immunity. Rather than cataloging miRNAs one by one, this review organizes the evidence around the major barriers that sustain an immune-cold CRC microenvironment: altered checkpoint and costimulatory signaling, defective antigen presentation, impaired effector T-cell access and function, suppressive myeloid and stromal compartments, and extracellular vesicle (EV)-mediated intercellular communication. We also critically assess tissue and circulating miRNA signatures as candidate biomarkers of ICI response and discuss therapeutic approaches based on miRNA mimics, inhibitors, and targeted delivery platforms. The available evidence supports a biologically compelling role for miRNA networks in CRC immune regulation, but clinical translation remains limited by context dependence, delivery, off-target effects, and the lack of treatment-linked validation in CRC cohorts.

## 1. Introduction

Colorectal cancer (CRC) is the second leading cause of cancer-related death and the third most commonly diagnosed cancer worldwide [1]. Outcomes have improved over time, aided by earlier diagnosis and advances in care [2], but metastatic CRC remains difficult to cure, and treatment-related morbidity remains substantial [3,4,5].

Immune checkpoint inhibitors (ICIs) targeting the programmed cell death protein 1 (PD-1)/programmed cell death ligand 1 (PD-L1) axis and cytotoxic T-lymphocyte-associated protein 4 (CTLA-4) can restore antitumor T-cell activity and disrupt immunosuppressive signaling within the tumor microenvironment (TME) [6]. The U.S. Food and Drug Administration (FDA) has approved pembrolizumab for first-line treatment of unresectable or metastatic microsatellite instability-high (MSI-H) or mismatch repair-deficient (dMMR) CRC, nivolumab plus ipilimumab for unresectable or metastatic MSI-H/dMMR CRC, and single-agent nivolumab for previously treated MSI-H/dMMR metastatic CRC [7,8]. Dostarlimab also has a tissue-agnostic indication for previously treated dMMR recurrent or advanced solid tumors without satisfactory alternatives [9]. These successes, however, apply to a minority of patients. MSI-H/dMMR tumors represent about 15% of CRC overall and approximately 5% of advanced metastatic disease [10], whereas most CRC is microsatellite-stable/mismatch repair-proficient (MSS/pMMR), a setting in which ICI response rates have generally remained below 10% [11].

The biological contrast between these disease states is central to immunotherapy response. MSI-H/dMMR tumors typically have high tumor mutational burden (TMB), abundant frameshift-derived neoantigens, and a pre-existing inflamed or “hot” TME with substantial CD8^+^ T-cell infiltration. MSS/pMMR tumors more often have low TMB, fewer neoantigens, impaired antigen-presentation programs, and a poorly inflamed or immune-excluded TME enriched in cancer-associated fibroblasts (CAFs), myeloid-derived suppressor cells (MDSCs), and regulatory T cells (Tregs) [12]. Accordingly, microsatellite and mismatch-repair status remain established biomarkers of ICI sensitivity [13].

For clarity, we use “inflamed” or “hot” for tumors with intratumoral effector T cells and active inflammatory signaling; “immune-excluded” for tumors in which immune cells accumulate in stromal or invasive-margin compartments but poorly penetrate tumor nests; and “immune-desert” or “cold” for tumors with sparse effector T-cell infiltration [14,15]. When using the broader term “immune-cold state,” we refer operationally to CRC microenvironments with ineffective antitumor immunity, including immune-desert and immune-excluded patterns.

These categories are useful but not absolute. Objective responses are not universal even in MSI-H/dMMR CRC [16,17,18], and immune-enriched MSS/pMMR organoid models with greater immune infiltration show greater sensitivity to PD-1 inhibition [19]. Likewise, immune-checkpoint expression varies substantially within MSI-H/dMMR tumors, and some tumors with low checkpoint-metagene expression resemble MSS/pMMR disease [20]. Thus, mismatch-repair status provides an essential clinical framework, but the depth and organization of immune infiltration remain important determinants of response. Understanding the regulators of these features is therefore relevant to both biomarker development and efforts to extend ICI benefit in MSS/pMMR CRC.

MicroRNAs (miRNAs) are short (~22-nucleotide), single-stranded, noncoding RNAs that participate in post-transcriptional gene regulation [21]. Because target recognition requires only partial sequence complementarity, one miRNA can influence multiple transcripts and, in turn, entire regulatory programs [22]. MiRNAs are broadly linked to human disease and are being pursued as diagnostic and therapeutic targets [23]. Increasing evidence also links miRNAs to immune checkpoint expression, T-cell responses, suppressive immune-cell states, and vesicle-mediated communication within the TME [24,25,26,27]. This network-level reach makes miRNAs plausible contributors to the coordinated immune-cold phenotype of MSS/pMMR CRC, while also making their effects highly context-dependent.

The central question for this review is therefore not simply which miRNAs are altered in CRC, but which miRNA circuits are most relevant to antitumor immunity, ICI response, and therapeutic reprogramming. We focus on tumor-intrinsic, immune-cell, stromal, and EV-mediated mechanisms; distinguish mechanistic evidence from association; and evaluate whether reported miRNA signatures are sufficiently treatment-linked to support biomarker claims.

Several recent reviews provide complementary perspectives. Sen et al. discuss miRNAs as bidirectional regulators of cold-to-hot conversion and chemoresistance; Kennel and Greten synthesize the broader CRC immune microenvironment; Pająk et al. emphasize diagnostic and prognostic miRNA markers; Longo et al. focus on CRC pathogenesis and therapeutic implications; Shirzad et al. systematically catalog CRC-associated miRNAs; and Włodarczyk et al. cover CRC and colitis-associated cancer across pathogenesis, diagnosis, and treatment [14,28,29,30,31,32]. The present review differs by organizing the evidence around immune-resistance functions in MSS/pMMR CRC and by asking how strongly each miRNA axis is supported as a mechanistic driver, a biomarker, or a therapeutic target.

## 2. MiRNA Biology and Dysregulation in CRC

### 2.1. MiRNA Biogenesis and Mechanisms of Action

**Figure 1:**
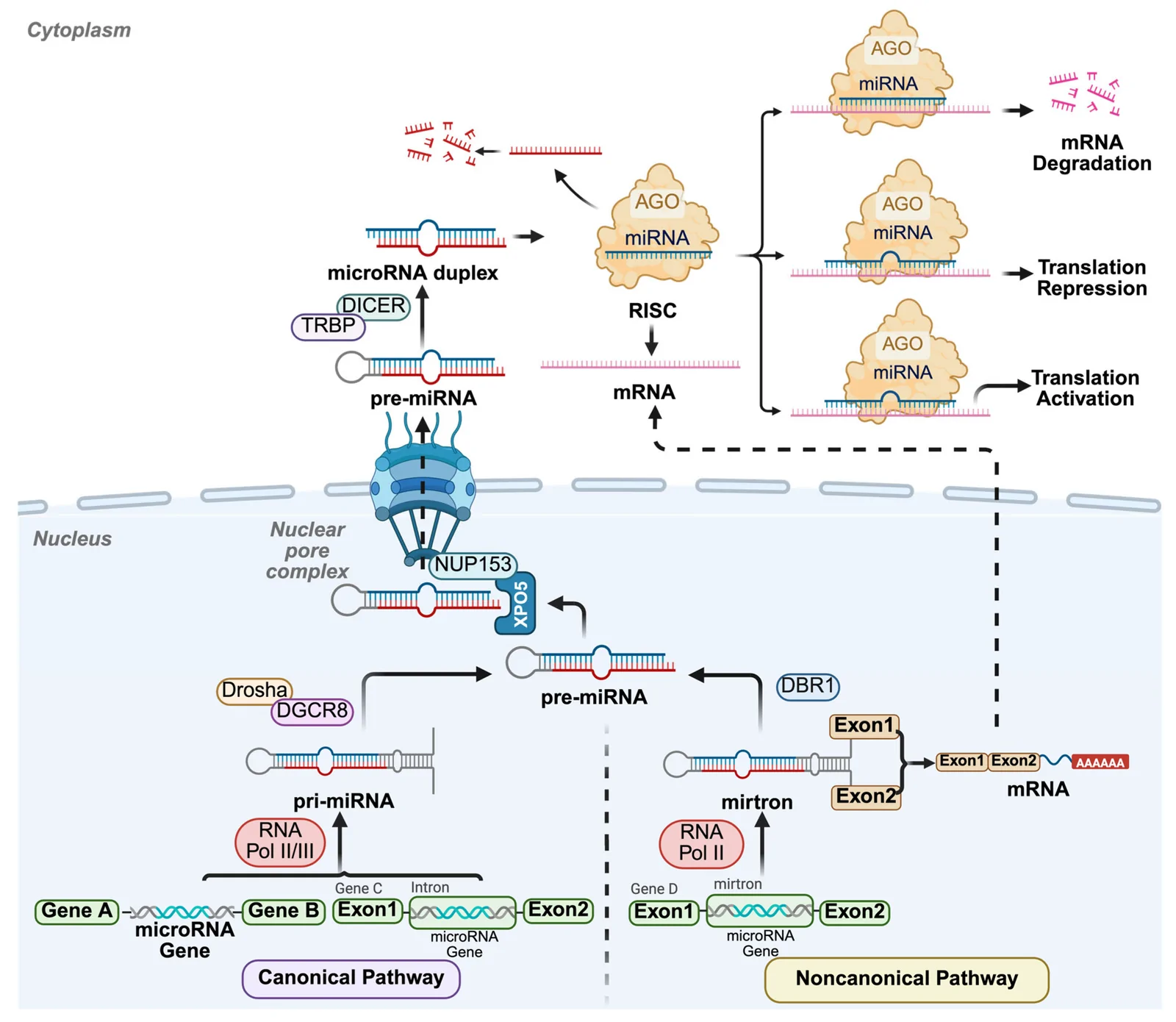
Biogenesis and Mechanisms of Action of MiRNAs. In the canonical pathway, miRNA genes located in intergenic or intronic regions are transcribed by RNA polymerase II or III into primary miRNAs (pri-miRNAs), which are processed in the nucleus by the DROSHA-DGCR8 Microprocessor complex into precursor miRNA (pre-miRNA) hairpins. In the noncanonical mirtron pathway, short intronic hairpins are excised by the spliceosome rather than by the Microprocessor and converge on the same pre-miRNA intermediate. Exportin-5 (XPO5) transports pre-miRNAs through the nuclear pore complex into the cytoplasm, where DICER, assisted by transactivation-response element RNA-binding protein (TRBP), cleaves the pre-miRNA near the terminal loop to generate an approximately 22-nucleotide miRNA duplex. The guide strand is loaded into Argonaute (AGO) to form the RNA-induced silencing complex (RISC), whereas the passenger strand is typically released and degraded. Guided by seed-region pairing to target messenger RNAs, RISC can direct mRNA degradation or translational repression and, in selected contexts, can activate translation. Abbreviations shown in the figure: DBR1, RNA lariat debranching enzyme 1; NUP153, nucleoporin 153. (Created with BioRender.com).

MiRNA biogenesis proceeds through coordinated nuclear and cytoplasmic processing steps (Figure 1). Canonical primary miRNA transcripts are processed in the nucleus by the Microprocessor complex containing DROSHA and DiGeorge syndrome critical region 8 (DGCR8), exported to the cytoplasm, further processed by DICER, and incorporated into Argonaute-containing RNA-induced silencing complexes (RISC) [33,34]. Target recognition depends largely on pairing through the miRNA seed region, typically nucleotides 2–8 from the 5′ end of the guide strand [35]. Once engaged, RISC can reduce target messenger RNA (mRNA) stability or inhibit translation. Figure 1 summarizes the canonical and noncanonical pathways relevant to the discussion below.

Disruption of the biogenesis machinery can itself reshape the CRC miRNA landscape. Exportin-5 (XPO5) is overexpressed in CRC in The Cancer Genome Atlas (TCGA) dataset and in an independent cohort of 246 clinical samples [36]. Its expression increases with stage in MSS tumors, and experimental inhibition suppresses tumor-cell growth and reduces several oncogenic miRNAs. The KH-type splicing regulatory protein (KSRP), another component of miRNA maturation, can be functionally perturbed by the oncogenic KAI1 C-terminal interacting tetraspanin (KITENIN) complex in highly metastatic CRC cells, resulting in impaired production of multiple tumor-suppressive miRNAs (tsmiRs) [37]. These studies illustrate that global changes in miRNA output can arise upstream of any single miRNA-target interaction.

### 2.2. MiRNA Dysregulation in CRC

CRC develops through interacting genetic and epigenetic alterations, and miRNA dysregulation should be viewed within that broader regulatory context [38]. MiRNA profiles correlate with microsatellite status, stage, tissue compartment, and other molecular features, but association alone does not establish predictive value for ICI response. Treatment-linked validation is therefore essential when discussing these signatures as immunotherapy biomarkers.

Representative human CRC tissue-profiling studies are summarized in Table 1. Paired tumor-normal analyses identify distinct miRNA programs and stage-associated changes, compartment-resolved profiling shows that cancer glands and surrounding stroma can carry different miRNA signatures, and colorectal liver metastases display miRNA and miRNA-mRNA patterns distinct from adjacent nonmalignant liver, with associations with overall and recurrence-free survival [39,40,41]. The practical implication is that a bulk-tissue miRNA measurement can reflect both tumor-cell biology and the composition of the surrounding microenvironment.

Mechanistically, miRNA dysregulation in CRC can arise from oncogenic signaling, altered transcriptional and epigenetic control, competing endogenous RNA (ceRNA) networks, and microbial exposures. These mechanisms are not independent: they converge on tumor-cell state and tumor-immune communication, providing several routes by which miRNA networks can contribute to immune evasion.

#### 2.2.1. Genetic, Epigenetic, and Post-Transcriptional Regulation

Oncogenic drivers can alter miRNA expression and connect genetic lesions to epigenetic and immune phenotypes. In *KRAS*-mutant CRC cells, *KRAS*^G12D^ decreases miR-29b-3p, increasing DNA methyltransferase 3 beta (DNMT3B) expression, promoter hypermethylation, and epigenetic silencing of stimulator of interferon genes (STING) [42]. In *KRAS*- or *BRAF*-mutant CRC cells, c-MYC suppresses miR-22-3p and thereby increases PHD finger protein 8 (PHF8), which in turn reinforces c-MYC expression [43]. MYC also represses miR-26a/b, relieving repression of La-related protein 1 (LARP1) and supporting the translational machinery required for tumor biosynthesis [44]. Additional layers of regulation are provided by SET domain bifurcated histone lysine methyltransferase 1 (SETDB1), which represses miR-22 and promotes PD-L1 expression [45], and by activator protein 4 (AP4), which represses the *MIR22HG*/miR-22-3p locus and thereby alters the mediator of DNA damage checkpoint 1 (MDC1), chromosomal instability, and senescence [46].

Post-transcriptional competition adds another layer. The circular RNA circNCOA3 sequesters miR-203a-3p, derepresses C-X-C motif chemokine ligand 1 (CXCL1), recruits MDSCs, suppresses CD8^+^ T-cell activity, and promotes anti-PD-1 resistance in CRC tumors [47]. This example is particularly informative as it links a ceRNA circuit directly to an immune-cell phenotype and treatment resistance rather than only to tumor-cell proliferation.

#### 2.2.2. Microbial Reprogramming of Host MiRNA Expression

The intestinal microbiome provides an additional, sustained source of miRNA reprogramming [48]. *Fusobacterium nucleatum* (*F. nucleatum*) increases host miR-21 through Toll-like receptor 4 (TLR4)/myeloid differentiation primary response 88 (MYD88)/nuclear factor-κB (NF-κB) signaling [49], increases miR-155-5p and suppresses *MutS homolog 6* (*MSH6*) [50], and reduces miR-1322 with downstream C-C motif chemokine ligand 20 (CCL20) induction and M2-like macrophage polarization [51]. *F. nucleatum*-infected CRC cells also release exosomes enriched in miR-1246, miR-92b-3p, and miR-27a-3p together with chemokines that can propagate prometastatic and inflammatory signals [52]. Enterotoxigenic *Bacteroides fragilis* (ETBF) similarly alters host and vesicular miRNA signaling, including miR-149-3p-dependent effects on T helper 17 (Th17) differentiation [53]. These observations support a model in which microbial exposures can sustain miRNA-dependent changes in the CRC microenvironment rather than acting as isolated transient events.

### 2.3. Tumor-Intrinsic MiRNA Programs with Immune Relevance

Not every tumor-intrinsic miRNA phenotype is directly informative for immunotherapy, so we emphasize examples with a plausible bridge to immune function. MiR-124-3p suppresses PD-L1 and Treg induction while also reducing CRC-cell proliferation and inducing apoptosis [24], and mesenchymal stromal cell (MSC)-derived EV delivery of miR-15a intersects with the lysine demethylase 4B (KDM4B)/homeobox C4 (HOXC4)/PD-L1 pathway and suppresses immune escape [54]. Exosomal miR-372-5p raises macrophage PD-L1, resulting in suppressed CD8^+^ T-cell activity [26]. MiR-451a is also relevant because its restoration induced features of immunogenic cell death and modulated dendritic-cell (DC) maturation in CRC cell-line models, although the magnitude of these effects was cell-context dependent [55]. These examples illustrate why the immune consequence of a single miRNA cannot be inferred from a tumor-cell phenotype alone.

## 3. MiRNAs Across Molecular Subtypes and Immune Phenotypes

Microsatellite status and mismatch-repair proficiency provide the clinical backbone for understanding CRC immunogenicity. MSI-H/dMMR tumors generally have high TMB and abundant neoantigens, whereas MSS/pMMR tumors more often show low-TMB, immune-cold or immune-excluded phenotypes [10,11,12,13]. MiRNA expression also varies with mismatch-repair context, but most reported signatures remain associative or computational. The sections below therefore use molecular subtype as context while reserving predictive claims for evidence linked directly to treatment response.

### 3.1. Consensus Molecular Subtypes and Immune Phenotypes

In CRC, dMMR/MSI-H status remains a key determinant of eligibility for ICI therapy, whereas the consensus molecular subtype (CMS) framework provides additional transcriptional and stromal context [56,57]. CMS1 (MSI-immune) is typically hypermutated and microsatellite-unstable, with prominent cytotoxic T-lymphocyte and natural killer (NK)-cell infiltration, interferon-γ (IFN-γ) signaling, and an inflamed microenvironment. CMS2 (canonical) is predominantly epithelial and characterized by WNT/MYC activation and generally low immune infiltration, broadly resembling an immune-desert state. CMS3 (metabolic) is also predominantly epithelial but is distinguished by metabolic dysregulation and relatively limited immune infiltration. CMS4 (mesenchymal), in contrast, exhibits transforming growth factor beta (TGF-β) signaling and mesenchymal/stromal activation, together with angiogenic, inflammatory, and immunosuppressive programs, high fibroblast density, and enrichment of monocytic-lineage signatures [58]. Nevertheless, CMS4 tumors may retain intermediate CD8^+^ T-cell infiltration [58]; therefore, CMS4 should not be assumed to exhibit spatial exclusion of cytotoxic T cells solely on the basis of its molecular subtype. These relationships provide useful biological anchors, but CMS categories should not be treated as interchangeable with formally defined immune phenotypes.

### 3.2. MiRNA Signatures Related to CMS Subtypes and MSI Status

MiRNA profiling further resolves this molecular heterogeneity. MSI-H/dMMR and MSS/pMMR CRCs exhibit distinct miRNA-expression patterns [59,60], and individual miRNAs are associated with specific molecular features. Elevated miR-31 expression is strongly associated with *BRAF* mutation and lesions arising through the serrated pathway [61,62]. High tumor miR-31-5p expression has also been associated with shorter progression-free survival in patients with metastatic CRC receiving anti-epidermal growth factor receptor (EGFR) therapy [63]. At a broader level, genome-wide miRNA-expression profiles can distinguish Lynch syndrome-associated CRC, sporadic MSI CRC, and sporadic MSS CRC [64], while other studies have linked miRNA patterns to MSI status and additional CRC molecular phenotypes [65,66]. Epigenetic analyses have further identified hypermethylation at the *MIR345* and *MIR132* loci in dMMR CRC [67] and methylation-induced loss of miR-484 in MSI CRC [68]. Collectively, these findings establish associations between miRNA patterns and CRC molecular subtypes or treatment outcomes, but they do not demonstrate that these miRNAs predict benefit from immune checkpoint inhibitors.

That distinction is important when relating miRNA signatures to CMS biology. Most published miRNA datasets do not include explicit CMS annotation, so assigning individual miRNAs to immune-desert or immune-excluded subtypes often relies on pathway inference rather than direct measurement in subtype-annotated cohorts. MiRNAs involved in antigen presentation and DC priming may plausibly contribute to immune-desert states, whereas stromal and TGF-β-associated programs may align more closely with immune exclusion. At present, these relationships are best regarded as mechanistic hypotheses rather than validated CMS-specific predictive signatures.

### 3.3. MiRNAs, Mismatch Repair Machinery, and the Immune Microenvironment

MiRNAs can both influence and respond to mismatch repair (MMR) biology. MiR-155 represses key MMR genes, including *MutS homolog 2* (*MSH2*), *MSH6*, and *MutL homolog 1* (*MLH1*), and can promote mismatch-repair defects [50,69]. In inflammatory bowel disease, elevated miR-155 and miR-21 have been linked to MMR protein dysregulation [70]. MiR-21 targets *MSH2* and *MSH6*, whereas miR-1290 directly targets *MSH2*, and both have also been linked to fluorouracil resistance [71,72]. Conversely, estradiol/estrogen receptor β (ERβ) signaling has been linked to lower miR-135b, miR-31, and miR-155 expression and to preservation of MMR gene expression [73]. Genomic instability can, in turn, reshape the miRNA landscape, as some miRNA genes are themselves targets of microsatellite instability, with frameshift mutations reported in *MIR1273c*, *MIR1303*, and *MIR567* in MSI-H/dMMR CRC [74].

The MMR-miRNA relationship also intersects with immune regulation. In MSI-H/dMMR CRC, reduced miR-148a-3p increases surface PD-L1 and promotes immune evasion [75]. *F. nucleatum* positivity has been associated with MSI-H CRC [76], providing a potential microbial link to this molecular context. MiR-99a is more abundant in MSS/pMMR than in MSI-H/dMMR CRC, and lower miR-99a in MSI-H tumors is associated with increased mechanistic target of rapamycin (mTOR) signaling and a shifted T helper 1/T helper 2 (Th1/Th2) immune profile [77]. Collectively, these observations reinforce the need to interpret miRNA effects within a defined mismatch-repair and cellular context.

## 4. MiRNA Networks in CRC Immune Evasion

**Figure 2:**
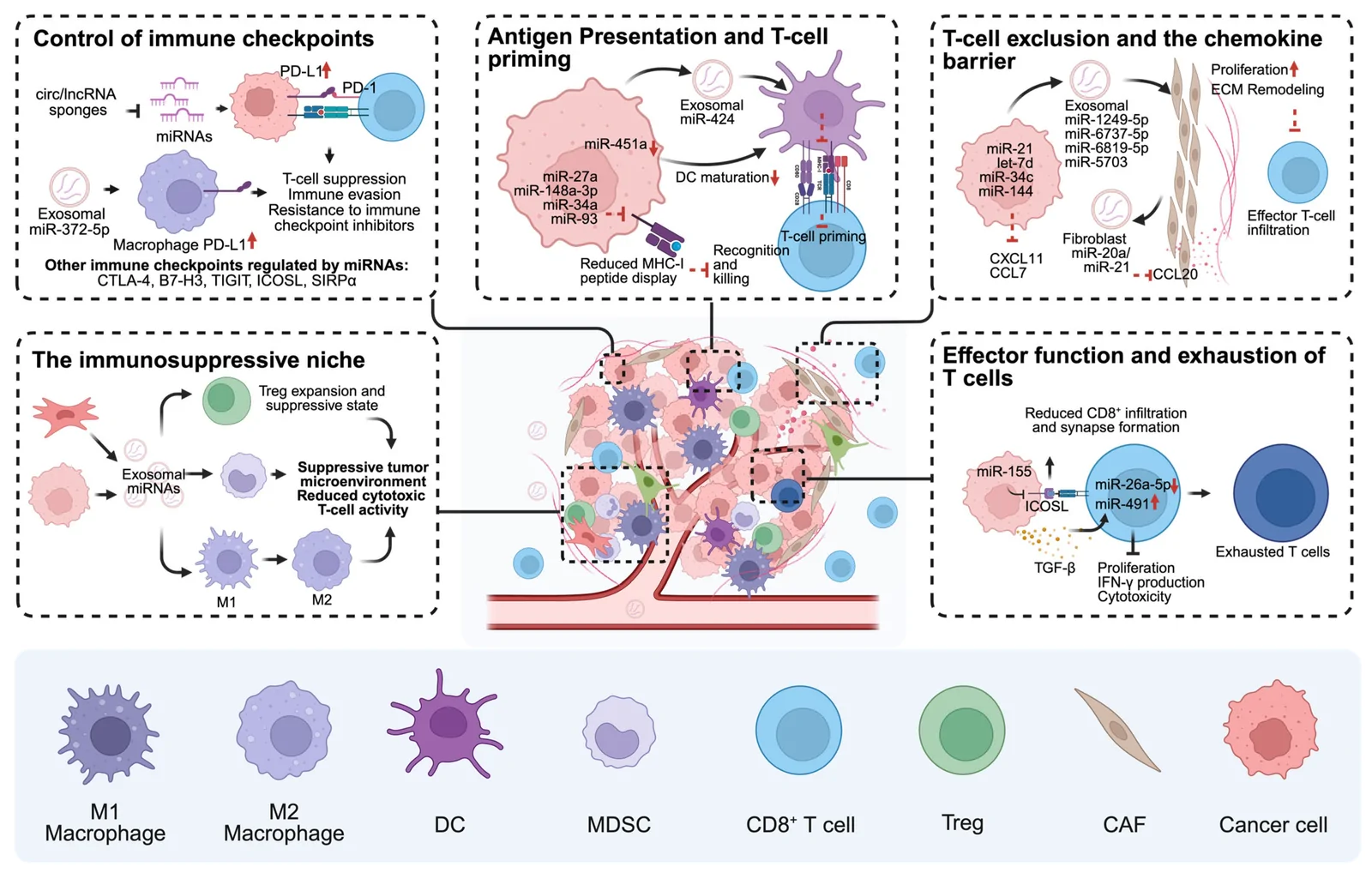
MiRNA Networks and the Tumor Microenvironment in CRC. The central panel shows a cross-section of the colorectal tumor microenvironment, with dashed boxes marking five determinants of the immune-cold phenotype, expanded in the surrounding panels. In immune checkpoint regulation, miRNAs and their noncoding RNA sponges alter PD-L1 and other checkpoint molecules to promote immune evasion and resistance to ICIs. In antigen presentation and T-cell priming, miRNAs repress MHC class I machinery and impair dendritic-cell maturation, weakening CD8^+^ T-cell recognition and priming. In T-cell exclusion and the chemokine barrier, miRNAs reprogram fibroblasts and suppress effector chemokines to limit T-cell infiltration. In effector dysfunction and exhaustion, miRNAs impair CD8^+^ T-cell cytotoxicity and interferon-γ production. In the immunosuppressive niche, EV-associated miRNAs expand Tregs and MDSCs and polarize macrophages toward an M2-like state. Red upward and downward arrows indicate increased and decreased expression, respectively, whereas blunt-ended lines indicate repression. Cell types are identified in the bottom box: DC, dendritic cell; Treg, regulatory T cell; CAF, cancer-associated fibroblast; MDSC, myeloid-derived suppressor cell; PD-L1, programmed cell death ligand 1; MHC-I, major histocompatibility complex class I; IFN-γ, interferon-γ; ICI, immune checkpoint inhibitor; TME, tumor microenvironment; B7-H3, B7 homolog 3; IDO1, indoleamine 2,3-dioxygenase 1; ICOSL, inducible T-cell co-stimulator ligand; SIRPα, signal regulatory protein alpha; TIGIT, T-cell immunoreceptor with immunoglobulin and immunoreceptor tyrosine-based inhibitory motif domains; CCL20, C-C motif chemokine ligand 20; CXCL11, C-X-C motif chemokine ligand 11; TGF-β, transforming growth factor beta. (Created with BioRender.com).

The CRC TME is not a collection of independent immune defects, but an interacting ecosystem of tumor cells, fibroblasts, endothelial cells, and immune populations. MiRNA networks can operate across these compartments, linking checkpoint regulation, antigen presentation, chemokine gradients, myeloid polarization, and T-cell function. EV-mediated transfer further allows a miRNA produced in one compartment to alter the behavior of another. Figure 2 summarizes this systems-level view of how miRNA dysregulation can reinforce immune exclusion, impaired T-cell surveillance, and resistance to ICI therapy.

### 4.1. Mechanisms of MiRNA-Mediated Immune Regulation

Because the literature spans very different experimental systems, we distinguish evidence levels throughout this section. Computational predictions are treated as hypothesis-generating; reporter assays and endogenous target-modulation studies as molecular evidence; xenograft or immunocompetent syngeneic studies as in vivo evidence; and human cohorts or interventional studies as translational or clinical evidence. This distinction is important because a validated 3′ untranslated region (3′-UTR) interaction does not necessarily establish that the same circuit is quantitatively important in a human tumor.

#### 4.1.1. Direct Post-Transcriptional Repression of Immune-Related Transcripts

Direct repression of immune-related transcripts is among the best-supported mechanisms of miRNA-mediated immune regulation in CRC (Table 2). Luciferase reporters carrying a candidate 3′-UTR are useful for establishing sequence-dependent targeting, but the most persuasive studies also demonstrate endogenous target modulation and functional consequences. PD-L1 is one of the strongest examples. In MSS/pMMR CRC cells, interleukin-17A (IL-17A) signaling through NF-κB/p65 and nuclear respiratory factor 1 (NRF1) represses miR-15b-5p. Restoring miR-15b-5p lowers PD-L1, increases stromal CD8^+^ T-cell infiltration, and resensitizes anti-PD-1-resistant tumors [78]. In MSI-H/dMMR CRC, miR-148a-3p represses IFN-γ-induced PD-L1 and reduces T-cell apoptosis in co-culture [75]. MiR-124-3p, miR-93-5p, and miR-138-5p have likewise been validated as direct PD-L1 regulators, with additional functional evidence from co-culture or xenograft models [24,79,80].

B7 homolog 3 (B7-H3; CD276) is an immune checkpoint associated with poorer outcomes in CRC and can exert either costimulatory or coinhibitory effects depending on context [81]. MiR-29a directly represses B7-H3 at the transcript level, and loss of miR-29a in CRC tumors leads to B7-H3 accumulation and enhanced invasion and migration [82]. TGF-β1-induced miR-155 can indirectly increase B7-H3 and B7 homolog 4 (B7-H4) by suppressing miR-143, thereby contributing to an immunosuppressive feedback loop [83]. MiR-187 and miR-128 have also been reported to target B7-H3 and suppress CRC-cell proliferation and migration when restored [84,85].

Direct miRNA regulation has also been reported for other immune checkpoints and immune-regulatory molecules. MiR-448 and miR-153 have been validated as direct repressors of indoleamine 2,3-dioxygenase 1 (IDO1). MiR-448 reduces IDO1-driven apoptosis of CD8^+^ T cells [25], while miR-153 also enhances T-cell killing in vitro and improves xenograft control when combined with chimeric antigen receptor T-cell (CAR-T) therapy [86]. In CRC, higher inducible T-cell costimulator (ICOS) expression has been associated with less advanced disease and improved survival, with ICOS primarily detected on CD4^+^ T cells and inducible T-cell costimulator ligand (ICOSL) on macrophages and tumor cells [87]. MiR-155 targets ICOSL, weakening T-cell recognition of tumor cells, and restoration of ICOSL expression in CRC cells increases cytotoxic T-cell infiltration and reduces tumor growth [88]. T-cell immunoreceptor with immunoglobulin and immunoreceptor tyrosine-based inhibitory motif domains (TIGIT) restrains cytotoxic T-cell and NK cell activity. Engineered miR-206-loaded tumor-derived exosomes lower TIGIT expression and enhance antitumor immune activity in CRC models [89]. CD47-signal regulatory protein alpha (SIRPα) signaling restrains macrophage phagocytosis. In addition to regulating PD-L1, miR-148a has been reported to bind the 3′-UTR of SIRPα and promote M1-like macrophage differentiation, shifting macrophages toward a more immunostimulatory state [75,90].

Direct repression has also been reported for costimulatory molecules. MiR-424 targets the 3′-UTR of CD28, a receptor that provides a key costimulatory signal for T-cell activation [91]. MiR-424 packaged in tumor-cell-derived EVs suppresses the CD28-CD80/CD86 costimulatory pathway in tumor-infiltrating T cells and dendritic cells (DCs). Conversely, allogeneic MC38-derived EVs lacking functional miR-424 are internalized by DCs and increase CD8^+^ T-cell infiltration in CT26 tumors [92].

These reporter-based studies establish molecular plausibility, but they also illustrate a recurrent limitation in the field. A seed match in a cloned 3′-UTR can produce a robust reporter effect even when the miRNA and target are not co-expressed at biologically relevant levels in the same tumor compartment. Strong mechanistic claims therefore require convergence among reporter data, endogenous expression, target modulation, functional assays, and, where possible, in vivo evidence. This evidentiary standard is particularly important when a miRNA is proposed as a therapeutic target.

**Table 2:** MiRNA Regulation of Immune Checkpoint and Costimulatory Molecules in CRC.

MiRNA	Target	Route of Regulation	Functional Outcome in CRC	Ref.
miR-15b-5p	PD-L1	Direct 3′-UTR binding	IL-17A/NRF1 represses miR-15b-5p, thereby increasing PD-L1; a mimic restores CD8^+^ T-cell infiltration and anti-PD-1 sensitivity.	[78]
miR-148a-3p	PD-L1	Direct 3′-UTR binding	Blocks IFN-γ-induced PD-L1 and reduces T-cell apoptosis.	[75]
miR-148a	SIRPα (CD47 axis)	Direct 3′-UTR binding	Promotes M1-like differentiation with increased CD86 and reduces macrophage recruitment.	[90]
miR-124-3p	PD-L1	Direct 3′-UTR binding	Lost in CRC. Mimic lowers PD-L1 via signal transducer and activator of transcription 3 (STAT3) and blocks Treg induction.	[24]
miR-93-5p	PD-L1	Direct 3′-UTR binding	Lowers PD-L1 and matrix metalloproteinases 1, 2, and 9 (MMP-1/-2/-9), reducing invasion and immune evasion.	[79]
miR-138-5p	PD-L1	Direct 3′-UTR binding	Lost in CRC and inversely correlated with PD-L1. Mimic suppresses tumor growth.	[80]
miR-497	PD-L1	Sponged by hsa_circ_0136666	Sequestration raises PD-L1, activating Tregs and driving immune escape.	[93]
miR-372-5p	PD-L1 in macrophages	Tumor-derived EV transfer	Induces macrophage PD-L1 via phosphatase and tensin homolog (PTEN)/AKT serine/threonine kinase (AKT)/NF-κB.	[26]
miR-21-5p, miR-200a	PD-L1 (M2 macrophage)	Tumor-derived EV transfer	Promote M2-like macrophage polarization and PD-L1 expression through PTEN/AKT serine/threonine kinase (AKT) and suppressor of cytokine signaling 1 (SOCS1)/signal transducer and activator of transcription 1 (STAT1) signaling.	[94]
miR-15a	PD-L1	MSC-derived EV transfer	Lowers PD-L1 via KDM4B/HOXC4 and blocks immune evasion.	[54]
miR-29a	B7-H3	Direct 3′-UTR binding	Lost in CRC. Loss raises B7-H3 and increases invasion and migration.	[82]
miR-155	B7-H3, B7-H4 (indirect)	Induced by TGF-β1; silences CCAAT/enhancer-binding protein beta (CEBPB) to suppress miR-143	Relieves miR-143-mediated repression of B7-H3 and B7-H4, reinforcing a TGF-β1-dependent feedback loop.	[83]
miR-155	ICOSL	Direct 3′-UTR binding	Lowers ICOSL and weakens T-cell recognition. ICOSL restoration increases cytotoxic T-cell infiltration.	[88]
miR-187, miR-128	B7-H3	Direct 3′-UTR binding	Restoration lowers B7-H3 and suppresses CRC-cell proliferation and migration.	[84,85]
miR-34a	B7-H3	Represses sirtuin 1 (SIRT1), releasing NF-κB p65	Raises B7-H3 and cytokine secretion; immune effects are context dependent.	[95]
miR-449a, miR-449b, miR-548au	B7-H3	Direct 3′-UTR binding (miR-34a seed)	Transduction lowers B7-H3 in CRC cells.	[96]
NS-MX3 (synthetic miR-34a, G → C at position 18)	B7-H3 and PD-L1	Engineered dual-target mimic	Suppresses both checkpoints and produces antitumor activity in CRC models.	[96]
miR-424	CD28, CD80	Direct 3′-UTR binding and tumor-derived EV transfer	Upregulated in CRC. EV transfer disrupts CD28-CD80/CD86 costimulation in T cells and DCs; miR-424-deficient EVs restore antitumor T-cell responses, limit tumor growth, and can enhance ICI efficacy in CRC models.	[91,92]
miR-448	IDO1	Direct 3′-UTR binding	Lowers IDO1 and rescues CD8^+^ T cells from IDO1-driven apoptosis.	[25]
miR-153	IDO1	Direct 3′-UTR binding	Lowers IFN-γ-induced IDO1 and improves CAR-T-cell killing and xenograft control.	[86]
miR-206	TIGIT	Engineered tumor-derived exosome delivery	Lowers TIGIT and enhances antitumor immune activity.	[89]

#### 4.1.2. CeRNA-Mediated Derepression of Immune-Regulatory Targets

CeRNA interactions add another level of context dependence because they alter the amount of miRNA available to its targets rather than changing the target transcript directly. Hsa_circ_0136666 sequesters miR-497, increases surface PD-L1, and promotes Treg-associated immune escape [93]. By contrast, E1A binding protein p300 (EP300)-driven circRERE sequesters miR-6837-3p, derepresses mitochondrial antiviral signaling protein (MAVS), activates type I interferon signaling, and enhances anti-PD-1 activity in immunocompetent mice [97]. The opposing immune outcomes emphasize that a ceRNA circuit should be interpreted through the function and cellular location of the derepressed target, not simply through the identity of the miRNA sponge.

#### 4.1.3. Indirect Action Through Transcription Factors and Signaling Nodes

The same principle applies when miRNAs act through transcription factors or signaling nodes. A miRNA should not be assigned an invariant oncogenic or tumor-suppressive label without specifying the target, cell type, and disease context. Apparent contradictions often reflect different regulatory architectures rather than irreproducible biology.

MiR-34a illustrates this context dependence. By inhibiting sirtuin 1 (SIRT1), miR-34a can activate NF-κB p65 and increase B7-H3 expression and cytokine secretion in CRC models [95]. Conversely, miR-34a-enriched tumor-derived exosomes reduced PD-L1 expression and exerted antiproliferative, proapoptotic, and antimigratory effects in CT26 CRC cells in vitro [98]. MiR-148a likewise shows compartment-dependent effects. It can impair CD8^+^ T-cell function through the calnexin (CANX)/major histocompatibility complex class I (MHC-I) axis [99], while in other contexts it suppresses tumor-cell migration, modulates apoptosis and angiogenesis under hypoxia, or alters macrophage recruitment through SIRPα signaling [90,100,101].

For therapeutic development, this context dependence is not a semantic issue. It determines both efficacy and safety. MiR-34a, for example, can produce different phenotypes depending on p53 status, NF-κB activity, oxygen tension, and macrophage content. Similarly, the net effect of miR-148a depends on which target and compartment dominate in a given model. MiRNA mimics or inhibitors should therefore be evaluated in systems that retain the relevant immune and stromal components rather than in tumor cells alone.

#### 4.1.4. Extracellular Vesicle-Mediated Intercellular MiRNA Transfer

EV transport extends miRNA activity beyond the cell that produces it. Once delivered to a recipient cell, EV-associated miRNAs use the same post-transcriptional machinery as endogenous miRNAs, but the biological consequence depends on which cells release the vesicles, which cells take them up, and what other cargo accompanies the miRNA. In CRC models, tumor-derived EV miRNAs can promote M2-like macrophage polarization, expand Tregs, reprogram fibroblasts, and increase checkpoint expression [26,102,103,104]. Microbial signals can also reshape vesicular miRNA cargo and influence T-helper differentiation [53]. EVs should therefore be viewed primarily as a delivery route that connects otherwise separate compartments of the TME.

The same route can potentially be exploited therapeutically. MiR-34a-enriched CT26 tumor-derived exosomes increase CD8^+^ T-cell activity and IFN-γ secretion while reducing interleukin-6 (IL-6), IL-17A, and TGF-β in tumor-bearing mice [105]. MiR-155-enriched tumor exosomes can stimulate DCs, increase cytotoxic and helper T-cell infiltration, and reduce Treg abundance [106]. These studies support proof of principle for cargo engineering, although they remain preclinical and do not resolve the manufacturing and targeting challenges of therapeutic EVs.

### 4.2. Immune Functions Regulated by MiRNAs

The major immune barriers in CRC, such as defective antigen processing and presentation, dysregulated checkpoint and costimulatory signaling, chemokine-mediated T-cell exclusion, effector-cell dysfunction, and suppressive stromal and myeloid programs, are biologically coupled rather than independent. MiRNAs are well suited to participate in this coupling because one miRNA can regulate several nodes, and EV transfer can connect different cellular compartments. Table 3 summarizes representative EV-associated and non-EV mechanisms across these immune functions. The key implication is that an immune-cold MSS/pMMR tumor is better viewed as a coordinated state maintained by reinforcing barriers than as a single checkpoint defect.

#### 4.2.1. Antigen Processing, Presentation, and T-Cell Priming

Defects in MHC-I antigen processing and presentation are established immune-evasion mechanisms in CRC, although their relationship to ICI resistance is context-dependent, particularly in MSI-H/dMMR disease [131,132]. MiRNAs can impair MHC-I presentation by repressing endoplasmic reticulum (ER) chaperones required for peptide loading and MHC-I assembly or by altering regulators of antigen-presentation machinery. MiR-27a represses calreticulin, an ER chaperone involved in peptide loading, whereas miR-148a-3p represses calnexin, which is required for MHC-I folding and assembly. Both effects reduce MHC-I surface expression on CRC cells [99,107]. Neoantigens are also important determinants of T-cell recognition; one study found that candidate genes encoding tumor-specific neoantigens in CRC were associated with immune-cell infiltration and linked to multiple candidate regulatory miRNAs [133].

Higher stromal miR-34a and miR-93 expression has been associated with lower signal transducer and activator of transcription 1 (STAT1) and interferon regulatory factor 1 (IRF1) expression and an immune-quiescent CRC microenvironment [109]. A recent multi-omics analysis of metastatic CRC linked miR-4746 and miR-3677 to immune-evasion networks associated with endothelin receptor type B (EDNRB) and high mobility group box 1 (HMGB1) [134].

DCs capture tumor antigens and provide antigen-presentation and costimulatory signals required for T-cell priming. MiR-424 in tumor-derived EVs suppresses the CD28-CD80/CD86 costimulatory pathway in both DCs and T cells, removing a key second signal required for T-cell priming even when antigen is presented [91]. MiR-552 directly represses atypical chemokine receptor 4 (ACKR4) in human CRC cells, and the loss of tumor-cell ACKR4 impairs DC migration to tumor-draining lymph nodes, weakens T-cell priming, and reduces ICI sensitivity in immunocompetent CRC models [135].

#### 4.2.2. Immune Checkpoint and Costimulatory Pathways

Checkpoint and costimulatory pathways are summarized in Table 2. The most convincing examples are experimentally tested axes with a defined target and functional consequence rather than large computational target sets. The literature also extends beyond PD-1/PD-L1. IDO1, CD47, ICOSL, TIGIT, and related nodes place miRNA regulation at several stages of immune recognition and effector control. Associations such as the reduction of PD-L1, CD47, and CD39 after docosahexaenoic acid treatment, together with lower miR-424, miR-133a, and miR-142, are potentially informative, but they should be distinguished from direct target-validation studies [136].

#### 4.2.3. T-Cell Exclusion and the Chemokine Barrier

T-cell access to the tumor is governed in part by chemokine gradients, and several miRNA circuits converge on this barrier. Protein tyrosine phosphatase 1B (PTP1B) increases PD-L1 through a forkhead box O1 (FOXO1)/miR-34c/c-MYC axis and, through a separate mechanism, suppresses C-X-C motif chemokine ligand 11 (CXCL11), thereby limiting CD8^+^ T-cell recruitment [110]. Separately, reduced miR-144 is associated with increased CXCL11, and miR-144 directly targets the CXCL11 3′-UTR [111]. CRC-cell exosomes containing let-7d altered fibroblast C-C motif chemokine ligand 7 (CCL7) secretion and C-C motif chemokine receptor 2-positive (CCR2^+^) monocytic-cell migration in vitro, although the contribution of let-7d was considered provisional [112]. These examples link miRNA regulation to the spatial organization of immune infiltration rather than simply to immune-cell abundance.

CAFs are particularly relevant because they can create both physical and chemokine-mediated barriers to lymphocyte entry. In fibrotic CRC, thrombospondin-2 (THBS2)-expressing matrix CAFs at the tumor front restrain DC- and macrophage-derived C-X-C motif chemokine ligands 9 and 10 (CXCL9 and CXCL10), limiting C-X-C chemokine receptor 3-positive (CXCR3^+^) CD8^+^ T-cell recruitment. Fibroblast-specific THBS2 loss disrupts this exclusionary barrier and sensitizes tumors to checkpoint inhibition [137].

Tumor-derived EV-associated noncoding RNAs provide additional routes for fibroblast reprogramming. EV miRNAs from *TP53*-deficient CRC cells suppress fibroblast *TP53*, exosomal miR-5703 suppresses apelin receptor (APJ) and contributes to the acquisition of myofibroblast-like features, and hypoxic tumor-derived exosomal HIF1A antisense RNA 2 (HIF1A-AS2) sequesters miR-33, promoting CAF activation, angiogenesis, and extracellular-matrix remodeling [104,113,114]. Stromal miR-20a regulates fibroblast C-X-C motif chemokine ligand 8 (CXCL8) secretion [115]. MiR-21 and CCL20 are both upregulated in the CRC microenvironment but are predominantly localized to tumor-associated fibroblasts and infiltrating immune cells, respectively, making direct in vivo miR-21–CCL20 regulation unlikely [116]. Taken together, these studies support fibroblasts as an important compartment in which miRNA signaling may reinforce immune exclusion.

#### 4.2.4. Effector Function and Exhaustion of T Cells

Infiltrating cytotoxic T lymphocytes can acquire an exhausted or dysfunctional phenotype within the immunosuppressive microenvironment. An in vivo clustered regularly interspaced short palindromic repeats (CRISPR)-based screen of tumor-antigen-specific CD8^+^ T cells in CRC identified miR-155 as important for both intratumoral CD8^+^ T-cell accumulation and response to anti-PD-1 therapy [118]. In CD8^+^ T cells, miR-155 enhances C-X-C chemokine receptor 6 (CXCR6) expression and effector function, whereas miR-155 expressed by malignant cells can instead repress the costimulatory ligand ICOSL [88]. Tumor-derived signals also modulate T-cell miRNA expression and can directly impair T-cell function. MiR-26a-5p is expressed at lower levels in CD8^+^ tumor-infiltrating lymphocytes (TIL) than in normal T cells and has been reported to act through EP300, suppressing signaling through phosphoinositide 3-kinase (PI3K), AKT serine/threonine kinase (AKT), and mTOR, thereby reducing cytotoxicity [119]. Tumor-derived TGF-β induces miR-491 in infiltrating CD8^+^ T cells, which targets cyclin-dependent kinase 4 (CDK4), T-cell factor 1 (TCF-1), and B-cell lymphoma 2-like protein 1 (BCL2L1), thereby slowing proliferation, reducing IFN-γ production, and promoting apoptosis [120].

#### 4.2.5. The Immunosuppressive Niche

Single-cell analyses of human CRC demonstrate a suppressive immune microenvironment containing activated regulatory T-cell states, exhausted T-cell populations, and diverse tumor-associated macrophages [138]. Within this niche, miR-125b-5p can attenuate Treg suppressive function by directly targeting tumor necrosis factor receptor 2 (TNFR2) [123]. Delivery of miR-125b-5p through mesenchymal stromal cell-derived exosomes improves responses to PD-1 blockade, while miR-125b-5p-mediated TNFR2 suppression in tumor cells can enhance sensitivity to PD-L1 blockade in CRC models [121,122]. Exosomal miR-208b promotes Treg expansion by targeting programmed cell death 4 (PDCD4), whereas exosomal miR-27a-3p, miR-30a-5p, and miR-320c repress interferon regulatory factor 4 (IRF4), thereby opposing IRF4-driven transdifferentiation of Tregs into macrophage-like cells [103,124].

The macrophage compartment is shaped by several exosomal miRNAs that promote an immunosuppressive M2-like state, including exosomal miR-934 and miR-106a-5p, as well as exosomal circPOLQ acting through miR-379-3p [102,125,126]. Myeloid miR-34a helps maintain an antitumor myeloid state; its loss promotes M2-like macrophage polarization and increases intestinal/colon tumor burden [127,128]. Tumor-derived EV miR-21-5p and miR-200a promote M2 polarization and increase macrophage PD-L1, thereby reducing CD8^+^ T-cell activity [94]. Exosomal miR-372-5p similarly increases macrophage PD-L1, suppresses CD8^+^ T-cell activation, and lowers interleukin-2 (IL-2) levels [26]. Exosomal miR-1246 alters C-C motif chemokine ligand 2 (CCL2) signaling and disrupts CD8^+^ T-cell infiltration [129].

MDSCs are a clinically relevant component of the immunosuppressive niche. Host miR-155 can also promote tumor growth through an MDSC-dependent mechanism [139]. In a real-world cohort of patients with MSS metastatic CRC treated with vascular endothelial growth factor receptor (VEGFR) inhibitor fruquintinib plus PD-1 inhibitors, circulating total and polymorphonuclear MDSCs decreased after treatment, particularly among patients with partial response or stable disease [140]. Sequestration of miR-203a-3p by circNCOA3 relieves repression of CXCL1, increases MDSC recruitment, and suppresses CD8^+^ T-cell activity in anti-PD-1-resistant tumors [47]. Inhibition of oncogenic miRNAs (oncomiRs) such as miR-17 reduces MDSC burden and can reshape the CRC immune microenvironment [130].

## 5. MiRNAs as Biomarkers of ICI Response

Current clinical biomarkers for ICI therapy in CRC are dominated by MMR/microsatellite status and related measures of tumor immunogenicity. TMB and PD-L1 may provide additional information in selected settings but do not fully capture the dynamic, post-transcriptional regulation of tumor-immune crosstalk. MiRNAs are attractive in principle because they can be measured in tissue and biofluids and may reflect active regulatory programs. The critical issue, however, is not measurability but predictive validity. A miRNA signature should not be considered an ICI biomarker unless it is reproducible, adds information beyond established markers, and is validated in treatment-linked cohorts.

### 5.1. Tumor Tissue MiRNA Signatures

Tumor tissue offers the clearest opportunity to link miRNA abundance to local molecular and immune features, but treatment context remains essential [66]. In PD-1-resistant CRC, the circNCOA3/miR-203a-3p/CXCL1 circuit promotes MDSC recruitment, and disrupting the circuit restores sensitivity in mice [47]. A separate study of a peptide vaccine restricted by human leukocyte antigen A*2402 (HLA-A*2402) associated high miR-196b-5p together with low miR-378a-3p and miR-486-5p with better response [141]. The latter is treatment-linked but not ICI-specific and should therefore be viewed as evidence that tissue miRNAs can track therapeutic response rather than as validation of an ICI biomarker.

Many computational miRNA signatures and risk scores have been proposed to represent immune features or putative immunotherapy sensitivity, but most are derived from public datasets and lack independent validation in ICI-treated CRC cohorts. These models are useful for hypothesis generation, not for clinical prediction. The next step is independent validation with predefined treatment-response endpoints and direct comparison against established biomarkers.

### 5.2. Circulating MiRNAs

Circulating miRNAs offer practical advantages for longitudinal sampling, but the current CRC evidence is largely treatment-associated rather than ICI-predictive. The EXOsome and cell-free miRNAs of anti-EGFR ResistAnce (EXONERATE) study used cell-free and exosomal miRNA profiles to predict response depth and survival during first-line anti-EGFR therapy in metastatic CRC [142]. Elevated exosomal miR-106a-5p and miR-934 are associated with metastatic behavior or adverse outcomes [102,125], and circulating miR-652-3p has been linked to regorafenib benefit and resistance [143]. These studies support translational feasibility, but direct profiling in ICI-treated CRC cohorts remains insufficient for a validated predictive claim.

### 5.3. Integration of MiRNAs with Microbiome and Other Biomarkers

Integration with other biomarkers may ultimately be more informative than a stand-alone miRNA measurement. MiR-155 and miR-21 are linked to MMR-deficiency/MSI biology through suppression of MMR proteins [69,70], and the combination of high *Fusobacterium* DNA with high miR-21 stratifies survival in CRC tissue [49]. *Porphyromonas gingivalis* can also reprogram CRC-derived EV miRNA and protein cargo and promote immune evasion through autophagy-mediated STING degradation [144]. These observations provide a rationale for combined molecular-microbiome signatures, but whether such panels improve prediction of ICI response beyond established biomarkers remains to be demonstrated.

### 5.4. Challenges in Biomarker Translation

The gap between discovery and clinical use is largely methodological. Many proposed miRNA biomarkers are derived from a single public dataset or a small single-institution cohort, and independently derived signatures for the same endpoint often share few components. Pre-analytical variables, analytical platforms, normalization methods, and sample type further complicate comparison because the measured abundance of a given miRNA can differ across serum, plasma, tissue, and EV preparations.

Accordingly, the field still lacks a miRNA biomarker ready for routine use in CRC immunotherapy. Progress will require independent cohorts, predefined treatment-specific endpoints, standardized sample handling and analytical workflows, cross-platform reproducibility, and direct evidence that a miRNA signature adds clinically useful information beyond MMR/MSI status, TMB, PD-L1, or other established assays.

For ICI prediction specifically, diagnostic or prognostic performance outside immunotherapy should not be relabeled as evidence of treatment-predictive accuracy. Future studies should report treatment-specific discrimination metrics and compare miRNA panels head-to-head with MMR/MSI status, TMB, PD-L1, the Immunoscore, and circulating tumor DNA (ctDNA), where appropriate. EV studies require an additional layer of standardization because isolation, enrichment, and characterization differ across laboratories; transparent reporting and adherence to the Minimal Information for Studies of Extracellular Vesicles 2023 (MISEV2023) guidelines should improve reproducibility [145].

## 6. Therapeutic Strategies to Improve ICI Responsiveness

Therapeutically, miRNAs are attractive because they can perturb several immune-resistance nodes at once, but that same pleiotropy creates liabilities. Two broad strategies dominate: double-stranded mimics can restore tumor-suppressive miRNAs (tsmiRs), whereas single-stranded antisense inhibitors (antagomirs or antimiRs) can inhibit oncomiRs. The pharmacologic requirements differ. Antisense inhibitors must reach the cytoplasm and bind the intended miRNA, while mimics must be loaded productively into RISC. Seed-mediated off-target repression is an additional design concern for duplex small-RNA therapeutics [146]. In immunotherapy, an additional challenge is spatial control: immune activation that is beneficial within the tumor may be harmful systemically.

**Figure 3:**
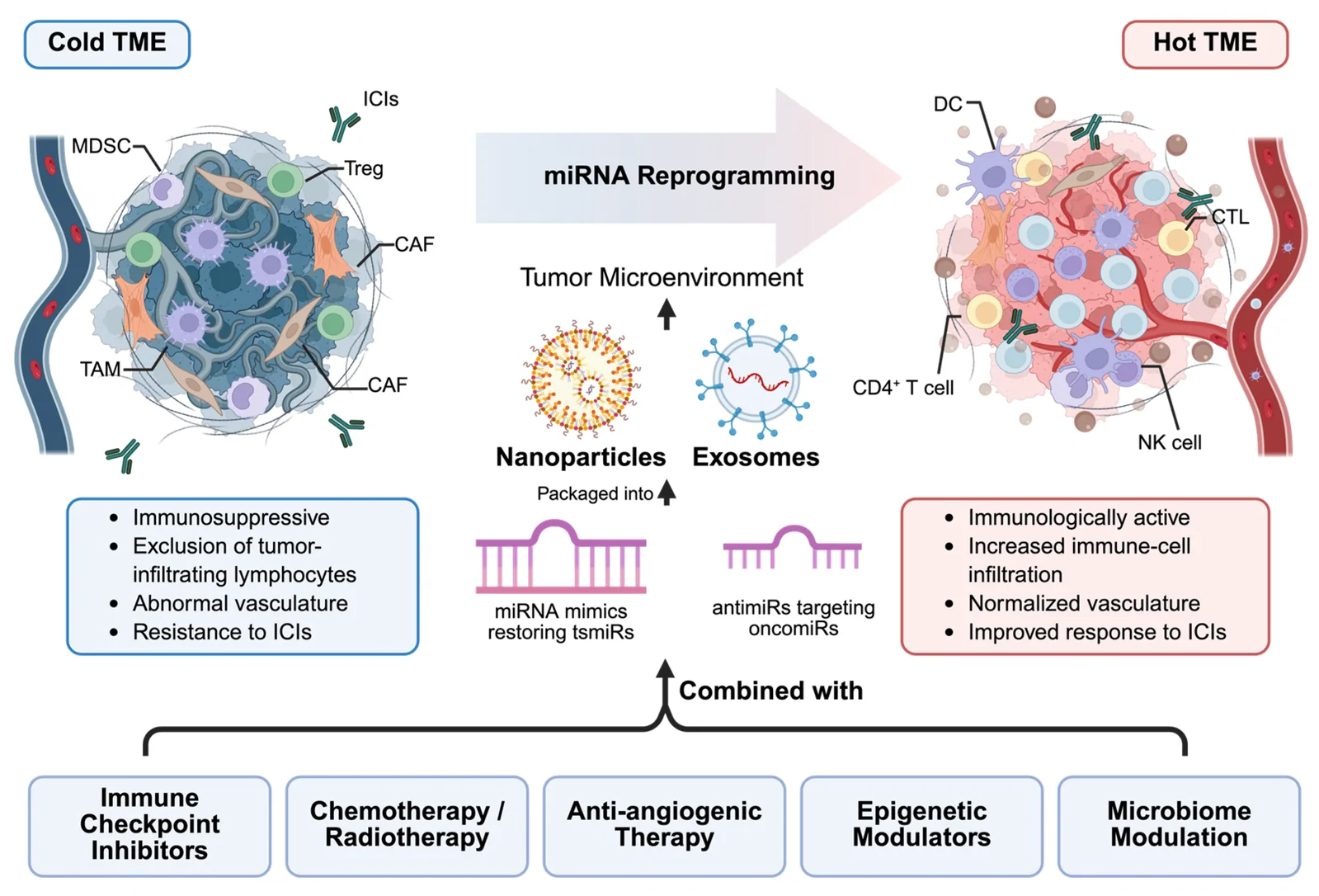
Therapeutic Reprogramming of the MiRNA Landscape to Convert an Immunologically “Cold” Colorectal TME into a “Hot,” Immunotherapy-Responsive State. The cold tumor microenvironment (TME; left) is immunosuppressive and is characterized by regulatory T cells (Tregs), myeloid-derived suppressor cells (MDSCs), tumor-associated macrophages (TAMs), cancer-associated fibroblasts (CAFs), exclusion of tumor-infiltrating lymphocytes, abnormal vasculature, and resistance to immune checkpoint inhibitors (ICIs). MiRNA mimics that restore tumor-suppressive miRNAs (tsmiRs) and antimiRs that inhibit oncogenic miRNAs (oncomiRs), delivered in nanoparticles or exosomes, can reprogram this microenvironment (center). The resulting hot TME (right) is more immunologically active, with greater infiltration by cytotoxic T lymphocytes (CTLs), CD4^+^ T cells, natural killer (NK) cells, and dendritic cells (DCs), more normalized vasculature, and improved responsiveness to ICIs. MiRNA-directed reprogramming may be combined with ICIs, chemotherapy or radiotherapy, anti-angiogenic therapy, epigenetic modulators, and microbiome-directed approaches to enhance immunotherapy sensitivity. (Created with BioRender.com).

Delivery chemistry therefore becomes part of the biology rather than a purely technical consideration. Unmodified oligonucleotides are unstable in circulation and are taken up inefficiently [147]. Phosphorothioate linkages, 2′-O-methyl and 2′-fluoro substitutions, and locked nucleic acid (LNA) residues can improve nuclease resistance and binding affinity [148], but chemical stabilization does not eliminate seed-mediated off-target repression [146]. For immune-cold CRC, the most credible therapeutic path likely combines mechanistically selected miRNA modulation with tumor-selective delivery and a partner therapy that addresses the same immune barrier (Figure 3).

### 6.1. Delivery Platforms

Several delivery platforms illustrate both the promise and the limitations of the field. Lipid nanoparticles (LNPs) are among the most clinically advanced systemic approaches. INT-1B3 is an LNP-formulated miR-193a-3p mimic that demonstrated tumor delivery and adaptive immune activation in preclinical models [149]. The program advanced to a first-in-human phase I/Ib study that enrolled 25 patients but was terminated because of insufficient funding [150]. MRX34, delivered in an amphoteric liposome, achieved tumor exposure and target modulation in humans but also highlighted the risk of systemic immune toxicity [151]. TargomiRs used targeted bacterial minicells and completed a phase I study in mesothelioma, establishing the feasibility of a non-lipid targeting strategy [152]. Engineered EVs are conceptually attractive because they exploit a natural transport pathway, although scalable manufacturing, cargo loading, and batch characterization remain unresolved. In MSS/pMMR CRC models, a pH-responsive solid lipid nanoparticle bearing PD-L1- and EGFR-binding peptides and an ER-homing peptide co-delivered a valosin-containing protein (VCP/p97) inhibitor, a PD-L1-targeting miR-142, and a Toll-like receptor 7 (TLR7) agonist [153]. Collectively, these studies establish delivery feasibility and pharmacologic proof of principle, but not yet clinical efficacy of miRNA therapy in CRC immunotherapy.

### 6.2. Combination with ICIs

Preclinical studies provide a clear rationale for combining miRNA modulation with checkpoint blockade. Reintroduction of miR-125b-5p improves the efficacy of anti-PD-L1 and anti-PD-1 therapy across CRC models [121,122], miR-15a reduces PD-L1, programmed cell death ligand 2 (PD-L2), and CTLA-4 while increasing antigen-presentation signals [154], and miR-148a-3p, miR-138-5p, and miR-140-3p suppress PD-L1 in relevant models [75,80,155]. Combined targeting of miR-21, miR-17, and miR-155 produces complementary tumor and systemic immune effects, including reduced MDSC burden [130], while N^6^-methyladenosine-modified circQSOX1 has been linked to anti-CTLA-4 resistance through Treg induction mediated by miR-326 and miR-330-5p sequestration [156]. The central limitation is model context: some syngeneic systems have relatively high TMB and are intrinsically ICI-responsive. Benefit in such models should not be assumed to predict conversion of clinically relevant MSS/pMMR tumors from cold to hot. Reproducibly demonstrating that transition is a more demanding and more informative preclinical benchmark.

### 6.3. Combination with Chemotherapy and Radiotherapy

Cytotoxic therapy can prime the immune system while simultaneously inducing compensatory resistance [157,158]. In a 22-patient pilot study of locally advanced MSS/pMMR CRC, neoadjuvant sintilimab with modified FOLFOX6 (mFOLFOX6; folinic acid, fluorouracil, and oxaliplatin) and bevacizumab produced substantial pathological regression, supporting the broader principle that cytotoxic, anti-angiogenic, and checkpoint-directed therapies can be combined to reshape the immune context [159]. MiRNA modulation offers a mechanistic extension of this approach. Inhibiting miR-27a increases oxaliplatin- or mitoxantrone-induced calreticulin exposure, along with adenosine triphosphate (ATP) and HMGB1 release, hallmarks of immunogenic cell death [108].

Co-delivery formulations extend this concept by packaging a cytotoxic agent together with an immune-modulating miRNA. A dual-loaded reconstituted high-density lipoprotein (HDL) nanocarrier containing α-hederin and oxaliplatin enhanced oxaliplatin sensitivity through Kelch-like ECH-associated protein 1 (KEAP1)/nuclear factor erythroid 2-related factor 2 (NRF2) signaling and was associated with a predicted miR-140-5p/PR domain zinc finger protein 1 (PRDM1) interaction, increased CD8^+^ T-cell activity, and improved antitumor responses in vivo [160].

Radiotherapy can promote immune priming but can also induce adaptive checkpoint resistance in CRC [157]. Restoring miR-29b-3p improved radiotherapy response, shifted the microenvironment toward greater immune infiltration, and enhanced ICI benefit [42]. In another preclinical approach, a PD-1 trap, a PD-L1-targeting miRNA, and Toll-like receptor 9 (TLR9)-inhibitory elements were packaged into an engineered adeno-associated virus (AAV) neoantigen-vaccine platform [161]. Combined with radiotherapy, this platform sustained neoantigen-specific cytotoxic T-lymphocyte responses, increased intratumoral T-cell infiltration, and eradicated tumors in mice.

In radioresistant CRC, tumor-derived miR-1226-5p suppresses IRF1 and is transferred to macrophages, where it promotes M2 polarization and TGF-β secretion; circSLC43A1 restrains this axis by acting as a sponge [162]. Drug repurposing may also reverse resistance through miRNA regulation. Tetrabenazine induces p53-dependent miR-34a-5p, lowers Snail expression, and reduces M2 macrophage polarization and interleukin-10 (IL-10) secretion in radioresistant CRC cells [163]. Together, these findings support further testing of miRNA mimics or antimiRs as components of chemo-radiotherapy combinations designed to amplify antitumor immunity.

### 6.4. Combination with Anti-Angiogenic Therapy

Vascular endothelial growth factor (VEGF)/VEGFR blockade can normalize tumor vasculature and alter immune-cell access. In CRC models, fruquintinib enhances anti-PD-1 activity by reducing angiogenesis, increasing intratumoral cytotoxic T-cell activity, and decreasing suppressive myeloid populations [164]. A phase II study of refractory pMMR/MSS metastatic CRC previously treated with bevacizumab also showed clinical activity of fruquintinib plus sintilimab [165]. MiRNA biology intersects with the same pathway. Exosomal miR-25-3p increases vascular endothelial growth factor receptor 2 (VEGFR2) expression, vascular permeability, and metastatic potential [166], whereas miR-148a suppresses hypoxia-associated proangiogenic signaling and miR-497 directly targets VEGF-A [101,167]. These observations provide a mechanistic rationale for combining miRNA modulation with anti-angiogenic/ICI strategies, but prospective testing in immunocompetent CRC models and clinical cohorts remains necessary.

### 6.5. Combination with Epigenetic Agents

Epigenetic therapy is relevant to miRNA-directed immunotherapy for two reasons: it can alter miRNA expression, and it can independently prime the TME. Dual DNA methyltransferase (DNMT)/histone deacetylase (HDAC) inhibition increases interferon signaling, dendritic-cell and T-cell infiltration, and anti-PD-L1 efficacy in immunocompetent CRC models [168]. DNMT/HDAC inhibition can also restore miR-9, miR-129, and miR-137, while other epigenetic interventions alter miR-143, miR-145, miR-133b, and miR-22 [169,170,171]. Dietary HDAC-modulating compounds such as sulforaphane and butyrate likewise influence miRNA programs [172,173]. Conversely, miRNAs can regulate epigenetic pathways. MiR-134-5p targets *KRAS* and *PIK3CA* while reducing HDAC expression, miR-148a-3p represses HDAC5, and miR-22 participates in feedback involving HDAC1, HDAC4, and SIRT1 [100,171,174]. The relationship is therefore bidirectional rather than a simple upstream-downstream hierarchy.

The translational relevance of this axis is supported by studies in MSS/pMMR CRC. In the randomized phase II CAPability-01 trial, adding the HDAC inhibitor chidamide to sintilimab, with or without bevacizumab, produced clinically meaningful responses and was associated with increased CD8^+^ T-cell infiltration [175]. In preclinical MSS CRC models, panobinostat combined with an IDO1-inhibitory nanodrug induced immunogenic cell death, activated DCs and CD8^+^ T cells, and reduced Tregs, MDSCs, and M2-like macrophages [176]. Butyrate has also been reported to improve anti-PD-1 efficacy by activating cytotoxic CD8^+^ T cells [177]. These data support epigenetic priming as a credible partner strategy, although the specific contribution of miRNA modulation to clinical benefit remains to be separated from the broader effects of epigenetic therapy.

### 6.6. Microbiome Modulation

The microbiome is another potential modifier of miRNA-regulated immunity. Host miRNAs can shape microbial communities, and gut bacteria can in turn reprogram host miRNA expression [48,178]. *F. nucleatum* alters miRNAs involved in chemokine and cytokine production, macrophage polarization, and chemotherapy sensitivity [51,52,179]. This bidirectionality makes the microbiome biologically interesting, but it also complicates causal interpretation because microbial composition, metabolites, host miRNAs, and immune state can change together.

Several mechanistic observations nevertheless connect microbial signals directly to immune resistance. Butyrate can counteract *F. nucleatum*-driven miR-130a-3p induction and downstream metabolic reprogramming [180]. *F. nucleatum*-derived succinic acid suppresses cyclic GMP-AMP synthase (cGAS)/interferon-β (IFN-β) signaling, limits CD8^+^ T-cell trafficking, and promotes resistance to anti-PD-1 therapy [181]. Through a distinct mechanism, the *F. nucleatum* Fap2 protein binds the inhibitory receptor TIGIT and suppresses NK- and T-cell activity [182]. These findings support the microbiome as an indirect lever for retuning the immune-cold state, but microbiome-directed approaches should be tested with the same treatment-linked and mechanistic rigor applied to miRNA biomarkers and therapeutics.

### 6.7. Challenges in Therapeutic Translation

No miRNA therapeutic has yet undergone an interventional trial specifically in CRC. This gap reflects more than a shortage of candidate miRNAs. Unmodified oligonucleotides degrade rapidly and are taken up inefficiently, while chemical modification and formulation alter stability, biodistribution, cellular uptake, and target engagement [147,148]. For CRC immunotherapy, tumor-selective delivery and direct pharmacodynamic evidence of target engagement should therefore be treated as core development requirements rather than optional optimization steps.

Off-target and off-tissue effects are equally important. Six- to eight-nucleotide seed matches can occur in many unintended transcripts, and most immune-regulatory miRNAs are not tumor restricted [146]. A miRNA that suppresses Tregs within a tumor, for example, may also perturb Tregs in normal tissues where they maintain self-tolerance. The termination of the MRX34 program after serious immune-mediated adverse events underscores the clinical importance of therapeutic index and spatial selectivity [151]. The field will need tumor-directed or tumor-conditional designs that preserve local immune reprogramming while limiting systemic immune toxicity.

## 7. Future Directions

### 7.1. Single-Cell and Spatial Resolution of Compartment-Specific MiRNA Activity

Many apparent contradictions in miRNA biology are likely to be resolved only when expression and function are mapped at cellular and spatial resolution. Single-cell miRNA profiling could establish whether a recurrent miRNA signal originates from tumor cells, fibroblasts, myeloid cells, or lymphocytes rather than assigning one function to the bulk tumor. Spatial methods could then relate miRNA activity to the invasive margin, fibroblast barriers, and immune-cell neighborhoods, allowing immune-excluded and immune-desert states to be tested directly. This level of resolution is also essential for designing delivery systems that target the compartment in which a miRNA is functionally relevant.

### 7.2. Multi-Omics Integration

MiRNA regulation is inherently multilayered, and direct repression of one transcript can propagate through signaling, transcriptional, metabolic, and epigenetic networks. Integrating miRNA profiles with matched transcriptomic, proteomic, epigenomic, and metabolomic data should help distinguish regulatory relationships from simple sequence complementarity. Such integration is especially important for ceRNA networks and immune-cold phenotypes, where the observable state likely arises from several interacting circuits rather than one dominant miRNA.

### 7.3. Machine Learning for Biomarker Discovery

Machine-learning approaches are increasingly being used for CRC miRNA biomarker discovery. A recent study combined fecal EV miRNA signatures with machine learning for noninvasive CRC detection and exploratory prognostic assessment [183]. This work illustrates the current direction of the field, but it also highlights an important distinction: most miRNA-based machine-learning studies address diagnosis or prognosis, not prediction of ICI response. Noninvasive sampling is attractive, yet prospective external validation and standardized pre-analytical workflows remain prerequisites for clinical use.

The specific application of miRNA-based machine learning to CRC immunotherapy remains underdeveloped. Most published ICI-response models use mRNA, broader transcriptomic, imaging, or multimodal features rather than miRNA profiles. The relevant test is therefore not whether a model can be trained, but whether miRNA information improves prediction in CRC cohorts with documented ICI outcomes beyond mismatch-repair status, TMB, PD-L1, and other established assays.

## 8. Conclusions

The accumulated evidence places miRNAs at multiple points in CRC antitumor immunity, where they regulate checkpoint expression, antigen presentation, chemokine signaling, T-cell function, myeloid and fibroblast states, and intercellular communication through EVs. This breadth makes miRNAs attractive as network regulators but also explains why their biological effects are strongly dependent on cell type, target availability, and disease context. At present, the evidence is strongest at the mechanistic and preclinical levels. No miRNA biomarker is in routine use for CRC immunotherapy, and no miRNA therapeutic has been tested in an interventional trial specifically in CRC.

The next phase of the field should therefore emphasize validation rather than accumulation of additional associations. Retrospective profiling of biospecimens from completed ICI trials in MSS/pMMR CRC could directly test whether defined miRNA states predict treatment response. Spatial and compartment-specific analyses should determine where recurrent miRNAs are expressed and which immune barriers they regulate. Therapeutic studies should prioritize tumor-selective delivery, pharmacodynamic target engagement, and models that faithfully represent immune-cold MSS/pMMR disease. EV studies should follow MISEV2023 guidance [145], and circulating-miRNA studies should use standardized pre-analytical and analytical workflows with prespecified validation plans.

A realistic translational goal is not to identify a universal “immunotherapy miRNA,” but to define a limited set of context-specific circuits that can either stratify immune states or be manipulated safely in combination with established therapies. Particularly testable directions include fibroblast- and myeloid-focused interventions in CMS4 CRC, tumor-conditional miRNA delivery, and miRNA-based priming before checkpoint blockade. Progress will depend on connecting precise mechanisms to the right cellular compartment, the right patient population, and a measurable treatment endpoint.

## Figures and Tables

**Table 1:** Representative MiRNA Profiling Studies in CRC Tissue.

Study	Cohort and Design	Comparison	Key Findings	Ref.
Shaath et al., 2021	15 paired CRC and adjacent non-tumor tissue samples analyzed by miRNA sequencing (miRNA-seq)	Tumor vs. adjacent normal; stage III/IV vs. stage I/II	Elevated in CRC tissue: miR-135b-5p, miR-552-5p, miR-552-3p, miR-224-5p, miR-183-5p. Decreased in CRC tissue: miR-133a-3p, miR-363-3p, miR-145-5p, miR-195-3p. Stage-associated miRNAs: let-7b-5p, miR-30a-5p, miR-145-5p, miR-133a-3p, and others were higher in stage III/IV disease.	[39]
Sato et al., 2020	24 CRC patients; crypt isolation with reverse-transcription quantitative polymerase chain reaction (RT-qPCR) panel of 13 miRNAs	Isolated cancer gland cells and surrounding stromal cells vs. non-tumor tissue	Elevated in gland cells: miR-130a-3p, miR-143-3p, miR-206, miR-31-5p, miR-27a-3p, miR-27b-3p. Decreased in gland cells: miR-21-5p, miR-195-5p, miR-19a-3p, miR-34b-3p, miR-186-5p, miR-191-5p, let-7a-5p. Patterns differ between gland and stromal compartments.	[40]
Rao et al., 2026	36 paired specimens of metachronous colorectal liver metastases and adjacent nonmalignant liver tissue analyzed by RNA sequencing	Liver metastasis vs. adjacent nonmalignant liver	108 upregulated and 92 downregulated miRNAs. Hub miRNAs included let-7c, miR-21-5p, miR-106a-5p, miR-139-5p, miR-101-3p, and miR-20b-5p.	[41]

**Table 3:** Representative miRNA-Centered, EV-Associated, and Non-EV Mechanisms Regulating the Immune Microenvironment in CRC. (↓ indicates downregulation; ↑ indicates upregulation).

miRNA/Regulatory RNA Axis	Target/Axis	Primary Compartment	Effect on Antitumor Immunity	EV Status	Ref.
**Antigen Presentation and T-cell Priming**					
miR-27a	calreticulin	tumor cells	↓ surface MHC-I; inhibition can enhance drug-induced immunogenic cell death.	No	[107,108]
miR-148a-3p	calnexin	tumor cells	↓ surface MHC-I; inhibition restores MHC-I and CD8^+^ T-cell killing.	No	[99]
miR-34a, miR-93	STAT1, IRF1 (inverse association)	stromal compartment	Associated with lower STAT1/IRF1 and an immune-quiescent phenotype.	No	[109]
miR-424	CD28–CD80/CD86	DCs, T cells	↓ costimulation and priming; effect demonstrated in MSS CRC models.	Yes	[91]
miR-451a	Immunogenic cell death (ICD) signals: calreticulin (CALR), adenosine triphosphate (ATP), HMGB1	tumor cells	Induces features of immunogenic cell death and modulates DC maturation; effects vary by cell line.	No	[55]
**T-cell Exclusion and the Chemokine Barrier**					
miR-34c (PTP1B/FOXO1/miR-34c/c-MYC axis)	c-MYC/PD-L1; parallel PTP1B/CXCL11 effect	tumor cells	PTP1B increases PD-L1 through the FOXO1/miR-34c/c-MYC axis and, through a separate mechanism, suppresses CXCL11, thereby limiting CD8^+^ T-cell recruitment.	No	[110]
miR-144	CXCL11	tumor cells	Reduced miR-144 increases CXCL11; miR-144 directly targets the CXCL11 3′-UTR.	No	[111]
let-7d	fibroblast CCL7	CAF	Alters fibroblast C-C motif chemokine ligand 7 (CCL7) secretion and C-C motif chemokine receptor 2-positive (CCR2^+^) monocytic-cell migration in vitro; let-7d contribution remains provisional.	Yes	[112]
miR-1249-5p, miR-6737-5p, miR-6819-5p	fibroblast *TP53*	CAF	Suppress fibroblast *TP53* and promote fibroblast proliferation.	Yes	[104]
miR-5703	APJ	CAF	Suppresses APJ and contributes to the acquisition of myofibroblast-like features.	Yes	[113]
miR-33	hypoxia-inducible factor 1 alpha (HIF-1α; direct); Notch1 and extracellular signal-regulated kinase (ERK) downstream	CAF	Exosomal *HIF1A-AS2* sequesters miR-33, promoting CAF activation, angiogenesis, and matrix remodeling.	Yes	[114]
miR-20a	CXCL8	CAF	Regulates paracrine CXCL8 secretion	No	[115]
miR-21	CCL20 (cell-line interaction only)	CRC TME	miR-21 is enriched mainly in tumor-associated fibroblasts, whereas CCL20 is mainly localized to infiltrating immune cells; direct in vivo regulation is not established.	No	[116]
**Effector Function and Exhaustion of T Cells**					
miR-155 (in CD8^+^ T cells)	SH2-containing inositol 5′-phosphatase 1 (SHIP1)	CD8^+^ T cells	Increases accumulation, effector function, and anti-PD-1 response.	No	[117,118]
miR-155 (in tumor cells)	ICOSL	tumor cells	Reduces CD8^+^ T-cell infiltration and immune synapse formation (context-dependent).	No	[88]
miR-26a-5p	EP300	CD8^+^ TILs	Reduces PI3K/AKT/mTOR signaling and cytotoxicity.	No	[119]
miR-491	CDK4, TCF-1, BCL2L1	CD8^+^ T cells	Reduces proliferation and IFN-γ production; promotes apoptosis.	No	[120]
**The Immunosuppressive Niche**					
miR-125b-5p	TNFR2	Tregs/tumor cells	Attenuates Treg suppression and improves anti-PD-1/PD-L1 response in CRC models.	Yes for MSC-derived delivery	[121,122,123]
miR-208b	PDCD4	Treg	↑ Treg expansion.	Yes	[103]
miR-27a-3p, miR-30a-5p, miR-320c	IRF4	Treg	Repress IRF4, thereby opposing IRF4-driven transdifferentiation of Tregs into macrophage-like cells.	Yes	[124]
miR-934	PTEN	TAM	M2 polarization (PI3K/AKT).	Yes	[102]
miR-106a-5p	suppressor of cytokine signaling 6 (SOCS6)	TAM	M2 polarization (Janus kinase 2 [JAK2]/STAT3).	Yes	[125]
circPOLQ/miR-379-3p	IL-10, STAT3	TAM	M2 polarization.	Yes	[126]
miR-34a	antitumor myeloid state	macrophages	Loss promotes M2-like polarization and increases intestinal/colon tumor burden.	No	[127,128]
miR-21-5p, miR-200a	PTEN, AKT	TAM	Promotes M2 polarization and macrophage PD-L1; reduces CD8^+^ T-cell activity.	Yes	[94]
miR-372-5p	PTEN/AKT/NF-κB	macrophages	Increases PD-L1, reduces IL-2, and suppresses CD8^+^ T cells.	Yes	[26]
miR-1246	CCL2 signaling	macrophages	Disrupts CD8^+^ T-cell infiltration.	Yes	[129]
miR-203a-3p (via circNCOA3)	CXCL1	MDSC	↑ MDSC recruitment; anti-PD-1 resistance.	No	[47]
miR-17	Not specified	MDSC	Inhibition reduces polymorphonuclear MDSC (PMN-MDSC) abundance.	No	[130]
**Microbial EV Signaling**					
miR-149-3p	Th17 differentiation	T cells (via *B. fragilis*)	Alters Th17 differentiation; microbial input.	Yes	[53]

## Data Availability

No new data were created or analyzed in this study. Data sharing is not applicable to this article.

## Abbreviations

The following abbreviations are used in this manuscript:

Abbreviation	Full term
3′-UTR	3′ untranslated region
AAV	Adeno-associated virus
ACKR4	Atypical chemokine receptor 4
AGO	Argonaute protein
AKT	AKT serine/threonine kinase
AP4	Activator protein 4
APJ	Apelin receptor
ATP	Adenosine triphosphate
B7-H3	B7 homolog 3
B7-H4	B7 homolog 4
BCL2L1	B-cell lymphoma 2-like protein 1
CAF	Cancer-associated fibroblast
CALR	Calreticulin
CANX	Calnexin
CAR-T	Chimeric antigen receptor T cell
CCL2	C-C motif chemokine ligand 2
CCL20	C-C motif chemokine ligand 20
CCL7	C-C motif chemokine ligand 7
CCR2	C-C motif chemokine receptor 2
CDK4	Cyclin-dependent kinase 4
CEBPB	CCAAT/enhancer-binding protein beta
ceRNA	Competing endogenous RNA
cGAS	Cyclic GMP-AMP synthase
CMS	Consensus molecular subtype
CRC	Colorectal cancer
CRISPR	Clustered regularly interspaced short palindromic repeats
ctDNA	Circulating tumor DNA
CTL	Cytotoxic T lymphocyte
CTLA-4	Cytotoxic T-lymphocyte-associated protein 4
CXCL1	C-X-C motif chemokine ligand 1
CXCL10	C-X-C motif chemokine ligand 10
CXCL11	C-X-C motif chemokine ligand 11
CXCL8	C-X-C motif chemokine ligand 8
CXCL9	C-X-C motif chemokine ligand 9
CXCR3	C-X-C motif chemokine receptor 3
CXCR6	C-X-C motif chemokine receptor 6
DBR1	RNA lariat debranching enzyme 1
DC	Dendritic cell
DGCR8	DiGeorge syndrome critical region 8
dMMR	Mismatch repair-deficient
DNMT	DNA methyltransferase
DNMT3B	DNA methyltransferase 3 beta
EDNRB	Endothelin receptor type B
EGFR	Epidermal growth factor receptor
EP300	E1A binding protein p300
ER	Endoplasmic reticulum
ERK	Extracellular signal-regulated kinase
ERβ	Estrogen receptor beta
ETBF	Enterotoxigenic *Bacteroides fragilis*
EV	Extracellular vesicle
EXONERATE	EXOsome and cell-free miRNAs of anti-EGFR ResistAnce
FDA	Food and Drug Administration
FOXO1	Forkhead box O1
HDAC	Histone deacetylase
HDL	High-density lipoprotein
HIF-1α	Hypoxia-inducible factor 1 alpha
HIF1A-AS2	HIF1A antisense RNA 2
HLA	Human leukocyte antigen
HMGB1	High mobility group box 1
HOXC4	Homeobox C4
ICD	Immunogenic cell death
ICI	Immune checkpoint inhibitor
ICOS	Inducible T-cell costimulator
ICOSL	Inducible T-cell costimulator ligand
IDO1	Indoleamine 2,3-dioxygenase 1
IFN-β	Interferon beta
IFN-γ	Interferon gamma
IL-10	Interleukin 10
IL-17A	Interleukin 17A
IL-2	Interleukin 2
IL-6	Interleukin 6
IRF1	Interferon regulatory factor 1
IRF4	Interferon regulatory factor 4
JAK2	Janus kinase 2
KDM4B	Lysine demethylase 4B
KEAP1	Kelch-like ECH-associated protein 1
KITENIN	KAI1 C-terminal interacting tetraspanin
KSRP	KH-type splicing regulatory protein
LARP1	La-related protein 1
LNA	Locked nucleic acid
LNP	Lipid nanoparticle
MAVS	Mitochondrial antiviral signaling protein
MDC1	Mediator of DNA damage checkpoint 1
MDSC	Myeloid-derived suppressor cell
mFOLFOX6	Modified folinic acid, fluorouracil, and oxaliplatin regimen
MHC-I	Major histocompatibility complex class I
miRNA	MicroRNA
miRNA-seq	MicroRNA sequencing
MISEV2023	Minimal Information for Studies of Extracellular Vesicles 2023
MLH1	MutL homolog 1
MMP	Matrix metalloproteinase
MMR	Mismatch repair
mRNA	Messenger RNA
MSC	Mesenchymal stromal cell
MSH2	MutS homolog 2
MSH6	MutS homolog 6
MSI	Microsatellite instability
MSI-H	Microsatellite instability-high
MSS	Microsatellite stable
mTOR	Mechanistic target of rapamycin
MYD88	Myeloid differentiation primary response 88
NF-κB	Nuclear factor-kappa B
NIH	National Institutes of Health
NK	Natural killer
NRF1	Nuclear respiratory factor 1
NRF2	Nuclear factor erythroid 2-related factor 2
NUP153	Nucleoporin 153
oncomiR	Oncogenic microRNA
PD-1	Programmed cell death protein 1
PD-L1	Programmed cell death ligand 1
PD-L2	Programmed cell death ligand 2
PDCD4	Programmed cell death 4
PHF8	PHD finger protein 8
PI3K	Phosphoinositide 3-kinase
pMMR	Mismatch repair-proficient
PMN-MDSC	Polymorphonuclear myeloid-derived suppressor cell
PRDM1	PR domain zinc finger protein 1
pre-miRNA	Precursor microRNA
pri-miRNA	Primary microRNA
PTEN	Phosphatase and tensin homolog
PTP1B	Protein tyrosine phosphatase 1B
RISC	RNA-induced silencing complex
RT-qPCR	Reverse-transcription quantitative polymerase chain reaction
SETDB1	SET domain bifurcated histone lysine methyltransferase 1
SHIP1	SH2-containing inositol 5′-phosphatase 1
SIRPα	Signal regulatory protein alpha
SIRT1	Sirtuin 1
SOCS1	Suppressor of cytokine signaling 1
SOCS6	Suppressor of cytokine signaling 6
STAT1	Signal transducer and activator of transcription 1
STAT3	Signal transducer and activator of transcription 3
STING	Stimulator of interferon genes
TAM	Tumor-associated macrophage
TCF-1	T-cell factor 1
TCGA	The Cancer Genome Atlas
TGF-β	Transforming growth factor beta
Th1	T helper 1
Th17	T helper 17
Th2	T helper 2
THBS2	Thrombospondin-2
TIGIT	T-cell immunoreceptor with immunoglobulin and immunoreceptor tyrosine-based inhibitory motif domains
TIL	Tumor-infiltrating lymphocyte
TLR4	Toll-like receptor 4
TLR7	Toll-like receptor 7
TLR9	Toll-like receptor 9
TMB	Tumor mutational burden
TME	Tumor microenvironment
TNFR2	Tumor necrosis factor receptor 2
TRBP	Transactivation-response element RNA-binding protein
Treg	Regulatory T cell
tsmiR	Tumor-suppressive microRNA
VA	U.S. Department of Veterans Affairs
VCP/p97	Valosin-containing protein
VEGF	Vascular endothelial growth factor
VEGFR	Vascular endothelial growth factor receptor
VEGFR2	Vascular endothelial growth factor receptor 2
XPO5	Exportin-5
